# Association between hyperuricemia and dietary retinol intake in Southwest China: a cross-sectional study based on CHNS database

**DOI:** 10.3389/fnut.2025.1508774

**Published:** 2025-01-22

**Authors:** Yi Liang, Tian Qiao, Xiaorong Ni, Lihui Yang, Tianhua Yao, Yiya Liu

**Affiliations:** ^1^Department of Clinical Nutrition, Affiliated Hospital of Guizhou Medical University, Guiyang, China; ^2^The Key Laboratory of Environmental Pollution Monitoring and Disease Control, Ministry of Education, School of Public Health, Guizhou Medical University, Guiyang, China; ^3^Guizhou Center for Disease Control and Prevention, Guiyang, China

**Keywords:** hyperuricemia, dietary retinol, cross-sectional design, CHNS, dose–response

## Abstract

**Background:**

Hyperuricemia is increasingly common in Southwestern China and poses significant health risks, including gout and cardiovascular disease. Retinol intake has been hypothesized to affect uric acid levels, but this relationship remains unclear.

**Methods:**

Data from 4,658 participants in the China Health and Nutrition Survey (CHNS) from 1991 to 2018 were analyzed. Dietary retinol intake was categorized using quantile-based methods, and hyperuricemia was identified as the primary outcome. Logistic regression models were used to estimate odds ratios and 95% confidence intervals, with analyses stratified by gender. Restricted cubic splines were utilized to evaluate the dose–response relationship.

**Results:**

The average age of participants was 40 ± 17.83 years, and 20.29% met the criteria for hyperuricemia. Logistic regression analysis identified a positive association between dietary retinol intake and hyperuricemia, with a more pronounced effect observed in men. The restricted cubic spline analysis revealed that the odds of hyperuricemia increased significantly when dietary retinol intake exceeded 3,538 IU/day for men and 4,504 IU/day for women.

**Conclusion:**

High dietary retinol intake is associated with an increased risk of hyperuricemia, with a stronger association observed in males. These findings suggest that dietary retinol intake under recommendation levels might be necessary to prevent hyperuricemia-related adverse health outcomes.

## 1 Introduction

Hyperuricemia, characterized by elevated serum uric acid levels, arises due to urate overproduction or its impaired excretion through the kidneys and gastrointestinal tract (1). The prevalence of hyperuricemia varies widely across populations, ranging from 8.9 to 24.4% (2–5). It serves as a major risk factor for gout (6) and is independently associated with chronic conditions, including type 2 diabetes (7), hypertension (8), metabolic syndrome (9), and chronic kidney disease (10). Various factors contribute to the onset of hyperuricemia, in addition to genetic predisposition and environmental influences, dietary habits like excessive alcohol consumption (11), high-purine diets (12), and the intake of high-fructose or sugary beverages (13) are well-established contributors to elevated serum uric acid levels. Additionally, previous studies have suggested potential associations between hyperuricemia and the intake levels of various vitamins, including vitamins C, D, E, and B1 (1, 14–16).

Retinol is an effective exogenous antioxidant and is believed to be involved in uric acid metabolism (17). Several studies have observed a positive correlation between serum retinol and uric acid levels (18–20), suggesting that higher retinol intake might elevate the risk of hyperuricemia. However, findings from retinol intake studies have been inconsistent. For instance, a study in Korea found lower dietary retinol intake in hyperuricemic individuals compared to controls (21), while a comparative study involving Australian and Norwegian cohorts reported a positive correlation between retinol intake and uric acid levels only in the Australian cohort (22). Furthermore, a cross-sectional survey in Taiwan found no significant association between retinol intake and hyperuricemia (23). These inconsistencies might be attributed to population-specific genetic variations in vitamin A metabolism (24), differences in overall dietary patterns affecting nutrient interactions (25), and varying environmental factors that influence vitamin bioavailability (26, 27). Such variations highlight the importance of population-specific investigations to better understand the relationship between retinol intake and hyperuricemia.

Given these inconsistent and limited findings on the relationship between dietary retinol intake and hyperuricemia, further research is warranted. This study aims to evaluate the relationship between dietary retinol intake and the risk of hyperuricemia in the population of Southwest China, using data from the China Health and Nutrition Survey (CHNS).

## 2 Methods

### 2.1 Study participants

The CHNS is an ongoing open-cohort longitudinal survey that has completed 10 rounds of data collection between 1989 and 2018. The survey employed a multi-stage random cluster sampling method across nine provinces in China, representing various levels of socioeconomic development. Each province was divided into counties and cities based on income levels (low, middle, and high), and a weighted sampling method was employed to randomly select four counties and two cities. Within the counties, villages and townships were randomly selected, while urban and suburban neighborhoods within the cities were also chosen at random. Finally, 20 households were randomly selected from each village, town, or community, and all household members participated in the CHNS interview. This survey aims to comprehensively capture key public health risk factors and health outcomes at the individual, household, and community levels, along with demographic, social, and economic variables. Since 2009, the CHNS has also collected geospatial coordinates of all respondents and key community resources, fasting blood samples from participants aged seven and above, and toenail samples from those aged two and above. Details have been described elsewhere (28). From 1991 to 2018, a total of 18,713 individuals participated in the survey, with 16,673 being adults aged 18 and above. For this study, we concentrated on 4,658 participants from Guizhou province who supplied detailed baseline information, completed dietary surveys, and provided blood samples.

The CHNS was approved by the institutional review boards of the University of North Carolina at Chapel Hill and the National Institute for Nutrition and Health, Chinese Center for Disease Control and Prevention. Written informed consent was obtained from all participants prior to data collection.

### 2.2 Dietary assessment

Dietary data were collected using a three-day 24-h recall method, combined with a food inventory method. Household food consumption was determined by examining the inventory changes from start to finish each day, using weighing and measuring techniques to ensure data accuracy. For foods that could not be directly weighed, estimates of wasted weight were used to compensate for missing data. A kitchen scale with a precision of grams was used for weighing food to ensure accurate measurements. Participants were thoroughly questioned about all food consumed in the past 24 h, whether eaten at home or away. The weight of individual food consumption was estimated using the household inventory method to calculate the total amount of each dish, multiplied by the individual’s reported consumption proportion, and further estimates of salt and oil intake were made. To exclude outliers, extreme dietary data were filtered based on the assessor’s professional judgment. The three-day recall method combined with the food inventory method used in this study showed a high correlation across food categories (29), enhancing the accuracy of dietary recall data. Energy and nutrient intake were calculated using the Chinese Food Composition Table (30).

### 2.3 Measurement of serum uric acid

Serum uric acid levels were measured using an enzymatic colorimetric method on a Hitachi 7,600 automated analyzer (Hitachi, Tokyo, Japan) with reagents from Randox Laboratories (Crumlin, United Kingdom). Hyperuricemia was defined as serum uric acid levels of ≥7 mg/dL in men and ≥ 6 mg/dL in women (31). Details on fasting blood sample collection have been described previously (32).

### 2.4 Covariates

Smoking was defined as the consumption of at least one cigarette per day. Alcohol consumption was assessed by asking participants if they had consumed beer or other alcoholic beverages in the past year, and responses were categorized as either ‘Yes’ or ‘No.’ Physical activity was measured across several domains: occupational (light, moderate, and vigorous), household (e.g., food preparation, shopping, laundry, child care), transportation (e.g., driving, walking, cycling), and leisure (e.g., yoga, dancing). Participants reported the average hours spent per week on these activities over the past year. Height and weight were measured according to World Health Organization (WHO) standards, with weight recorded to the nearest 0.01 kg and height to the nearest 0.1 cm. BMI was categorized into four groups: underweight (BMI < 18.5 kg/m^2^), normal weight (BMI 18.5–23.9 kg/m^2^), overweight (BMI 24.0–27.9 kg/m^2^), and obesity (BMI ≥ 28.0 kg/m^2^) (33). Blood pressure was measured three times using a mercury sphygmomanometer, and the average reading was recorded. Hypertension was defined as an average systolic pressure ≥ 140 mmHg, diastolic pressure ≥ 90 mmHg, self-reported hypertension, or current use of antihypertensive medication (34). Type 2 diabetes was defined as a fasting blood glucose level ≥ 7 mmol/L or an HbA1c level ≥ 6.5% (35).

### 2.5 Statistical analysis

We analyzed the distribution of sociodemographic characteristics, disease conditions, and dietary intake across quintiles of retinol consumption. Continuous variables were expressed as mean ± standard deviation and categorical variables were presented as frequencies and percentages. Logistic regression models were employed to assess the association between daily retinol intake and hyperuricemia. Models were adjusted for potential confounders, including age, sex, BMI, alcohol consumption, smoking status, physical activity, and comorbid conditions such as hypertension, diabetes, and chronic kidney disease. Odds ratios (ORs) and 95% confidence intervals (CIs) were reported. Subgroup analyses were performed to explore the potential effect modification by sex. Restricted cubic spline analysis was used to evaluate the dose–response relationship between dietary retinol intake and hyperuricemia. All tests were two-tailed, and *p* < 0.05 were considered statistically significant.

## 3 Results

### 3.1 Participant characteristics

The study included 4,658 participants with an average age of 40 ± 17.83 years, of whom 20.29% met the criteria for hyperuricemia. The sex-specific characteristics and dietary consumption of participants across quintiles of dietary retinol intake are summarized in Table 1. Males and females showed significant differences in age, BMI, waist circumference, alcohol intake, smoking status, physical activity, urbanization, hypertension, diabetes, and dietary intake of various nutrients.

**Table 1:** Sex-specific characteristics and dietary consumption of the CHNS of rentinol intake* (*n* = 4,658).

	Male			Female		
	Quintile 1	Quintile 3	Quintile 5	Quintile 1	Quintile 3	Quintile 5
Participants (n)	412	434	478	519	500	456
Characteristics						
Age (years)	45.11 (19.39)	40.48 (19.88)	44.21 (18.73)	49.10 (16.47)	46.74 (16.44)	44.30 (13.95)
BMI (kg/m^2^)	21.53 (3.26)	21.42 (3.81)	21.98 (3.70)	22.00 (3.39)	22.57 (3.64)	22.97 (3.10)
Waist (cm)	77.38 (10.59)	78.72 (12.14)	79.84 (11.00)	77.59 (10.35)	78.30 (11.38)	79.58 (10.91)
Alcohol intake (g/d)	0.00 (0.00)	5.04 (27.18)	0.49 (3.47)	0.00 (0.00)	1.46 (10.13)	0.13 (1.63)
Current smoking (%)	229 (16.65)	262 (19.05)	284 (20.65)	8 (28.57)	7 (25.00)	5 (17.86)
Physical activity (h/week)	33.49 (20.19)	38.86 (20.71)	33.67 (20.11)	34.23 (22.06)	29.08 (19.16)	29.22 (22.34)
Urban [n (%)]	56 (2.56)	121 (5.54)	115 (5.26)	83 (3.36)	154 (6.23)	120 (4.85)
Hypertension (%)						
Yes	41 (1.96)	18 (0.86)	25 (1.19)	27 (1.12)	27 (1.12)	20 (0.83)
Do not know	5 (0.24)	0 (0.00)	2 (0.10)	1 (0.04)	0 (0.00)	0 (0.00)
Diabetes (%)						
Yes	11 (0.58)	1 (0.05)	4 (0.21)	3 (0.14)	14 (0.64)	0 (0.00)
Do not know	4 (0.21)	0 (0.00)	2 (0.11)	1 (0.05)	0 (0.00)	0 (0.00)
Dietary intake						
Energy (kcal/day)	1734.40 (581.14)	1924.46(693.66)	2234.32 (701.18)	1548.66 (551.74)	1714.84 (688.15)	2294.37 (795.43)
Carbohydrate (g/day)	229.08 (94.96)	224.99 (88.82)	244.93 (107.59)	193.96 (74.00)	206.03 (107.65)	242.63 (119.24)
Fat intake (g/day)	66.05 (49.47)	84.80 (47.63)	109.21 (55.78)	64.69 (37.98)	75.36 (39.73)	116.44 (70.36)
Protein intake (g/day)	55.04 (23.84)	56.43 (21.30)	67.12 (24.82)	47.97 (22.44)	50.24 (19.15)	68.58 (28.00)
Fiber intake (g/day)	11.83 (13.56)	11.00 (6.66)	17.73 (9.61)	9.39 (8.55)	10.74 (6.45)	22.59 (15.19)
Cholesterol (mg)	109.90 (105.12)	244.69 (231.47)	275.80 (199.45)	132.46 (144.70)	183.07 (135.15)	256.41 (179.34)
Vitamin C (mg/day)	44.29 (30.60)	65.57 (36.87)	96.08 (41.31)	37.19 (25.58)	62.50 (34.44)	107.56 (54.48)
Vitamin E (mg/day)	25.33 (35.07)	27.38 (25.10)	27.86 (18.85)	22.65 (29.94)	23.67 (18.46)	30.57 (23.57)
Magnesium (mg/day)	223.37 (173.89)	188.80 (117.29)	254.65 (131.57)	176.63 (123.28)	178.55 (121.91)	273.53 (152.70)
Iron intake (mg/day)	20.27 (10.98)	21.68 (11.25)	25.89 (9.20)	16.15 (7.65)	18.17 (8.31)	26.55 (10.13)
Zinc intake (mg/day)	9.84 (4.30)	10.36 (3.91)	12.22 (4.70)	7.95 (3.41)	8.87 (3.46)	12.71 (4.72)
Vitamin A (µg RE)	72.82 (28.26)	355.09 (63.01)	1258.58 (494.74)	74.35 (31.81)	358.83 (66.56)	1338.52 (626.55)
Fish (g/day)	6.42 (26.79)	3.10 (15.57)	16.39 (43.98)	4.04 (16.67)	4.41 (19.81)	11.64 (32.70)
Dairy products (g/day)	7.39 (27.57)	14.64 (49.32)	7.90 (33.91)	4.59 (16.73)	7.98 (34.03)	4.86 (19.69)
Egg (g/day)	3.50 (8.40)	22.92 (34.55)	20.27 (27.69)	6.51 (9.89)	15.88 (20.87)	12.47 (19.11)
Dark vegetables (g/day)	7.58 (13.41)	43.68 (42.99)	118.30 (107.16)	8.69 (17.03)	50.70 (49.86)	116.91 (112.54)
Organ meats (g/day)	0.00 (0.00)	0.38 (4.56)	2.37 (9.88)	0.04 (0.91)	0.29 (3.50)	2.07 (9.09)

*Dietary data were collected using a three-day 24-h recall method, combined with a food inventory method.

### 3.2 Association between retinol intake and hyperuricemia

**Figure 1:**
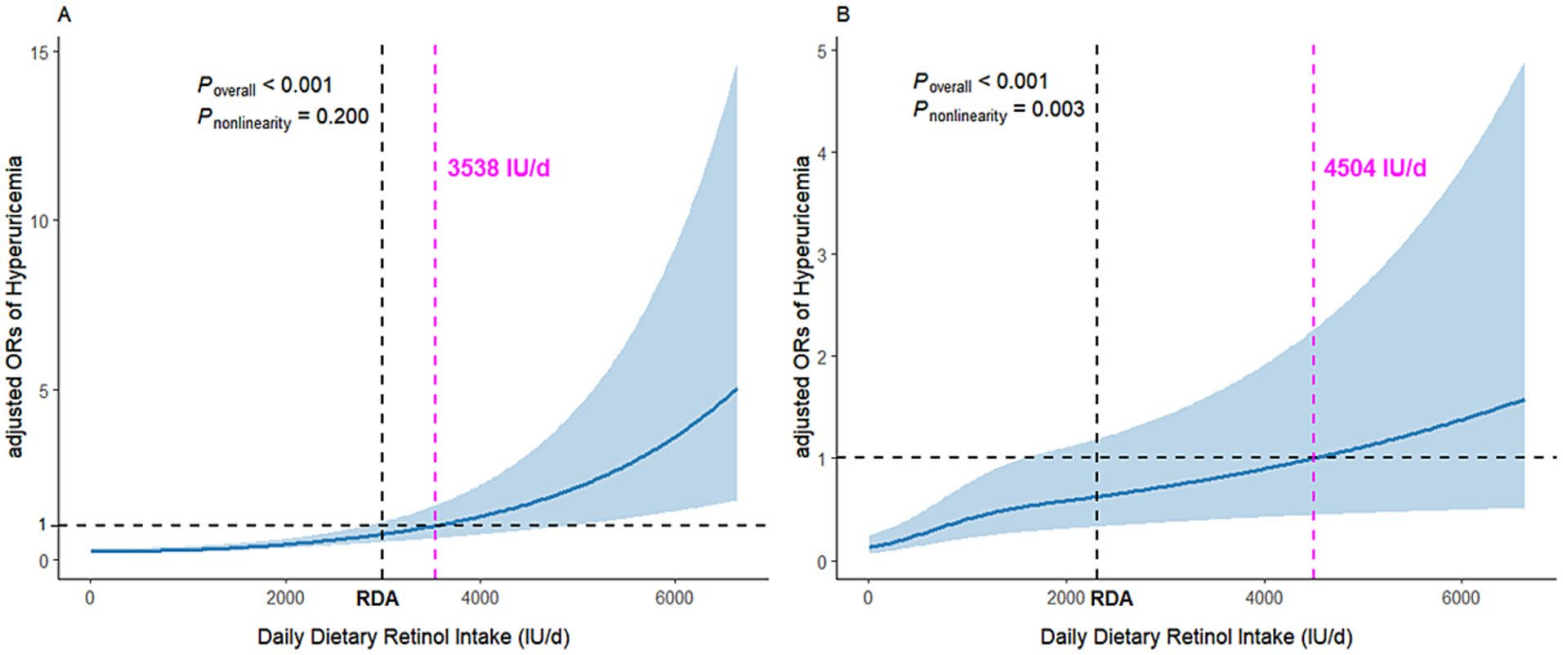
Dose–response relationship between dietary retinol intake and hyperuricemia. Panel **A** depicts data for men, while panel **B** shows data for women. The association was adjusted for age, residence, diabetes, BMI, educational level, smoking status, alcohol consumption, physical activity, total energy intake, fat (quintiles), dietary cholesterol (quintiles), dietary fiber (quintiles), and mutual adjustment for protein, and BMI (quintiles). The solid line and blue shading represent the estimated odds ratios (ORs) and their 95% confidence intervals (CIs).

The logistic regression analysis demonstrated a positive association between dietary retinol intake and the risk of hyperuricemia (Table 2). In males, the odds ratios (ORs) for hyperuricemia across increasing quintiles of retinol intake were 1.13 (95% CI: 0.81–1.58), 0.87 (95% CI: 0.62–1.22), 1.59 (95% CI: 1.17–2.16), and 2.31 (95% CI: 1.72–3.12), respectively, compared to the lowest quintile, with a significant trend (*p* < 0.0001) in the fully adjusted model. In females, the ORs were 1.14 (95% CI: 0.78–1.67), 1.49 (95% CI: 1.03–2.16), 2.00 (95% CI: 1.39–2.88), and 1.48 (95% CI: 1.01–2.16), respectively, with a significant trend (*p* = 0.0022) in the fully adjusted model. The restricted cubic spline analysis revealed a significant dose–response relationship between dietary retinol intake and the risk of hyperuricemia. The odds of hyperuricemia increased significantly when dietary retinol intake exceeded 3,538 IU/day for males and 4,504 IU/day for females (Figure 1).

**Table 2:** ORs (95% CIs) for hyperuricemia according to quintiles of dietary retinol intake.

	Quintile 1	Quintile 2	Quintile 3	Quintile 4	Quintile 5	*p*
Male						
Model 1	1.00	1.13 (0.81–1.58)	0.87 (0.62–1.22)	1.59 (1.17–2.16)	2.31 (1.72–3.12)	<0.0001
Model 2	1.00	1.17 (0.82–1.68)	0.87 (0.61–1.26)	1.58 (1.13–2.20)	2.28 (1.65–3.16)	<0.0001
Model 3	1.00	1.15 (0.81–1.64)	0.84 (0.59–1.21)	1.58 (1.13–2.19)	2.31 (1.67–3.18)	<0.0001
Model 4	1.00	1.17 (0.82–1.68)	0.87 (0.61–1.26)	1.58 (1.13–2.20)	2.28 (1.65–3.16)	<0.0001
Female						
Model 1	1.00	1.14 (0.78–1.67)	1.49 (1.03–2.16)	2.00 (1.39–2.88)	1.48 (1.01–2.16)	0.0016
Model 2	1.00	1.14 (0.78–1.67)	1.49 (1.03–2.16)	2.00 (1.39–2.88)	1.48 (1.01–2.16)	0.0012
Model 3	1.00	1.24 (0.82–1.86)	1.54 (1.03–2.30)	2.11 (1.43–3.12)	1.58 (1.05–2.37)	0.0018
Model 4	1.00	1.35 (0.89–2.05)	1.67 (1.10–2.52)	2.21 (1.48–3.31)	1.72 (1.13–2.61)	0.0022

Model 1: age (5-year category). Model 2: model 1 adjustments plus urban (yes or no), diabetes (yes or no), current smoking (never, past, and current), alcohol intake (quintiles), physical activity (quintiles), and total energy intake (quintiles). Model 3: model 2 adjustments plus fat (quintiles), dietary cholesterol (quintiles), dietary fiber (quintiles), and mutual adjustment for protein. Model 4: model 3 adjustments plus BMI (quintiles).

## 4 Discussion

Our study reveals that increased dietary retinol intake is linked to a higher prevalence of hyperuricemia, especially in men. Additionally, a J-shaped dose–response relationship between hyperuricemia and dietary retinol was observed. This suggests that excessive intake of retinol may potentially elevate uric acid levels. Previous studies exploring the relationship between retinol intake and hyperuricemia or serum uric acid levels relied on cross-sectional analyses. Data from the 1988–1994 NHANES highlighted a positive correlation between serum retinol levels and uric acid in the U.S. adult population (19). Similarly, research conducted among Korean adults robusted the results, demonstrating that higher retinol intake was associated with elevated uric acid levels (18, 36). Moreover, the use of isotretinoin, a retinoid medication for acne treatment, has been linked to increased uric acid levels, further supporting this relationship (37). The mechanistic link between retinol levels and hyperuricemia remains inadequately elucidated. One plausible explanation involves the role of elevated serum retinol in enhancing xanthine oxidase activity. This enzyme not only catalyzes the conversion of retinol to retinoic acid but also facilitates the oxidation of xanthine, leading to increased uric acid production (38, 39). However, to our knowledge, no prospective studies have yet explored the association between individual dietary retinol intake and uric acid levels or hyperuricemia.

The gender-stratified analysis highlights a pronounced susceptibility among men to hyperuricemia associated with elevated retinol intake. This finding aligns with established literature, which underscores the pivotal role of hormonal modulation in uric acid homeostasis. Specifically, prior studies have elucidated that estrogen facilitates uric acid excretion, thereby conferring a protective advantage against hyperuricemia in premenopausal women (40, 41). Conversely, testosterone has been implicated in upregulating uric acid synthesis, potentially exacerbating the risk of hyperuricemia in men (42). These results underscore the necessity for gender-specific dietary recommendations to mitigate the risk of hyperuricemia, particularly in populations with high retinol consumption.

Dose–response analysis revealed that exceeding the recommended dietary retinol intake (3,538 IU/day for men and 4,504 IU/day for women) was linked to a higher prevalence of hyperuricemia, regardless of dietary supplement use. These values notably exceed the current recommended daily intakes of 3,000 IU for men and 2,310 IU for women (43), highlighting the potential health risks of excessive consumption. These findings are consistent with previous studies indicating that excessive vitamin A can influence metabolic pathways related to uric acid production and excretion (44). Although hypervitaminosis A is uncommon under typical dietary conditions, the rising prevalence of nutritional supplements as a primary source of vitamins raises the possibility of inadvertent overconsumption. For instance, during the 2003–2006 NHANES survey, 53% of participants reported using dietary supplements, with 33% specifically consuming multivitamin/multimineral products that often contain retinol (45). However, this pattern differs significantly in our study population from Southwest China, a lower-income region where supplement use is minimal. According to the 2010–2012 CNHS survey, only 0.71% (95% CI: 0.49–0.94%) of the Chinese population aged 6 and older reported using nutritional supplements in the past month, with usage rates in impoverished areas as low as 0.09% (46). This low prevalence reflects both economic constraints and adherence to Chinese dietary guidelines, which emphasize obtaining nutrients primarily from food sources rather than supplements (47). Given the limited supplement use in our study population, we did not include this variable in the main analysis. While this aligns with local dietary patterns, the lack of comprehensive supplement use data represents a limitation that should be addressed in future research, particularly as supplement use patterns may evolve with economic development.

Our findings contribute to the growing body of evidence supporting a positive association between retinol intake and hyperuricemia. However, the relationship appears to vary across different populations, highlighting the importance of population-specific factors in this association. For instance, studies from different regions of China have yielded varying results: while research in northern China observed a positive correlation between serum vitamin A levels and uric acid concentrations in adults (48), a cross-sectional survey in Taiwan found no significant association between retinol intake and hyperuricemia (23). Similarly, a study conducted in Korea reported lower dietary retinol intake among individuals with hyperuricemia (21). These disparate findings may be partly explained by regional dietary habits. The distinctive dietary characteristics in Southwest China, characterized by lower consumption of vegetables, fruit, and animal products, along with a higher intake of grain and oil (49), may uniquely influence both retinol intake and uric acid metabolism. Such regional variations in dietary habits underscore the importance of considering local dietary contexts when developing nutritional guidelines for hyperuricemia prevention.

Additionally, results in the present study indicate that individuals with higher retinol intake frequently consume more dark vegetables, organ meats, and fish. These consumption habits possibly reflect broader lifestyle choices that influence hyperuricemia risk. Therefore, we adjusted for potential confounders, including physical activity, smoking, and alcohol consumption, to more accurately assess the relationship between retinol intake and hyperuricemia. On the other hand, understanding the influence of lifestyle factors on retinol intake and hyperuricemia is crucial for developing effective prevention strategies. Physical activity represents another critical lifestyle factor in this relationship, as regular exercise has been shown to help maintain healthy uric acid levels (50), whereas sedentary behavior may increase hyperuricemia risk (51). These findings suggest that managing hyperuricemia requires a comprehensive lifestyle approach. Indeed, previous research has demonstrated that combined interventions incorporating balanced dietary choices, regular physical activity, limited alcohol consumption, and weight management are more effective in preventing hyperuricemia than modifications to single factors (52). This integrated approach to lifestyle management may be particularly important for individuals with elevated retinol intake, as it involves multiple pathways that influence uric acid metabolism.

In summary, this study provides valuable insights into the association between dietary retinol intake and hyperuricemia within a Southwest China population, demonstrating a significant relationship through comprehensive dietary assessments and a large, representative sample. The dose–response analysis reveals specific threshold levels associated with increased risk, while gender-stratified results highlight the need for sex-specific recommendations. Despite methodological limitations inherent to our cross-sectional design and dietary assessment methods, which preclude causal inference and may introduce recall bias, these findings make a substantial contribution to our understanding of the retinol-hyperuricemia relationship. The regional dietary patterns characteristic of Southwest China, while potentially limiting broader generalizability, offer unique insights into population-specific nutritional factors affecting hyperuricemia. Future longitudinal and experimental studies are crucial to validate these observations and elucidate underlying mechanisms, particularly focusing on population-specific dietary patterns and their impact on hyperuricemia risk.

## Data Availability

The raw data supporting the conclusions of this article will be made available by the authors, without undue reservation.
